# Predictors of Multidrug- and Extensively Drug-Resistant Tuberculosis in a High HIV Prevalence Community

**DOI:** 10.1371/journal.pone.0015735

**Published:** 2010-12-29

**Authors:** Jason R. Andrews, N. Sarita Shah, Darren Weissman, Anthony P. Moll, Gerald Friedland, Neel R. Gandhi

**Affiliations:** 1 Division of Infectious Diseases, Massachusetts General Hospital, Harvard Medical School, Boston, Massachusetts, United States of America; 2 Departments of Medicine and Epidemiology & Public Health, Albert Einstein College of Medicine and Montefiore Medical Center, New York, New York, United States of America; 3 Department of Medicine, Albert Einstein College of Medicine and Montefiore Medical Center, New York, New York, United States of America; 4 Philanjalo and Church of Scotland Hospital, Tugela Ferry, KwaZulu-Natal, South Africa; 5 AIDS Program, Yale University School of Medicine, New Haven, Connecticut, United States of America; 6 Departments of Medicine and Epidemiology & Public Health, Albert Einstein College of Medicine and Montefiore Medical Center, New York, New York, United States of America; University of Delhi, India

## Abstract

**Background:**

Multidrug-resistant (MDR) and extensively drug-resistant (XDR) tuberculosis (TB) have emerged in high-HIV-prevalence settings, which generally lack laboratory infrastructure for diagnosing TB drug resistance. Even where available, inherent delays with current drug-susceptibility testing (DST) methods result in clinical deterioration and ongoing transmission of MDR and XDR-TB. Identifying clinical predictors of drug resistance may aid in risk stratification for earlier treatment and infection control.

**Methods:**

We performed a retrospective case-control study of patients with MDR (cases), XDR (cases) and drug-susceptible (controls) TB in a high-HIV-prevalence setting in South Africa to identify clinical and demographic risk factors for drug-resistant TB. Controls were selected in a 1∶1∶1 ratio and were not matched. We calculated odds ratios (OR) and performed multivariate logistic regression to identify independent predictors.

**Results:**

We enrolled 116, 123 and 139 patients with drug-susceptible, MDR, and XDR-TB. More than 85% in all three patient groups were HIV-infected. In multivariate analysis, MDR and XDR-TB were each strongly associated with history of TB treatment failure (adjusted OR 51.7 [CI 6.6-403.7] and 51.5 [CI 6.4–414.0], respectively) and hospitalization more than 14 days (aOR 3.8 [CI 1.1–13.3] and 6.1 [CI 1.8–21.0], respectively). Prior default from TB treatment was not a risk factor for MDR or XDR-TB. HIV was a risk factor for XDR (aOR 8.2, CI 1.3–52.6), but not MDR-TB. Comparing XDR with MDR-TB patients, the only significant risk factor for XDR-TB was HIV infection (aOR 5.3, CI 1.0–27.6).

**Discussion:**

In this high-HIV-prevalence and drug-resistant TB setting, a history of prolonged hospitalization and previous TB treatment failure were strong risk factors for both MDR and XDR-TB. Given high mortality observed among patients with HIV and drug-resistant TB co-infection, previously treated and hospitalized patients should be considered for empiric second-line TB therapy while awaiting confirmatory DST results in settings with a high-burden of MDR/XDR-TB.

## Introduction

Multidrug-resistant (MDR) and extensively drug-resistant (XDR) tuberculosis (TB) continue to emerge in high HIV prevalence settings, and their mortality in HIV co-infected patients remains high [1]–[5]. Despite a rising global awareness of drug-resistant TB, only 11% of the estimated cases of MDR TB were notified to the World Health Organization in 2008 [6]. Most cases likely go undetected due to insufficient laboratory infrastructure for diagnosis; in the vast majority of clinical settings in the developing world, culture and drug-susceptibility testing (DST) are not available. Even where DST is available, routine use in all TB suspects is often unfeasible due to limited sample processing capacity of laboratories and cost. Furthermore, the most commonly used culture and drug-susceptibility testing methodologies require six to eight weeks for results. In our studies of patients with MDR and XDR TB and HIV-co-infection, the majority of patients died within this time frame [4], [7]. Delays in diagnosis of drug-resistance also contribute to ongoing transmission [8]. Until laboratory infrastructure can be strengthened or rapid diagnostics made widely available, simple tools are needed to guide clinical decision-making in high HIV and drug-resistant TB prevalence settings.

Reliable assessment of a patient's risk of drug-resistant TB can enable targeting of DST where resources are limited. Further, it can aid clinicians in identifying patients for early initiation of second line anti-tuberculosis drugs while awaiting DST results. Indeed, an approach of early, aggressive management of drug-resistant TB has been shown to contribute to good patient outcomes and higher cure rates [9], [10]. Risk stratifying patients based on easily and rapidly-available clinical and/or laboratory data is commonly used in developed and developing-world settings for medical decision-making for a myriad of diseases [11]–[14]. However, few data have been available from high HIV- and TB-prevalent, resource-poor settings, where simple clinical tools to identify patients at high-risk for drug-resistant TB are most needed.

While several studies have shown previous TB treatment to be a risk factor for MDR TB [15], [16], little is known about the relative contribution of this and other risk factors in high HIV prevalence settings. Drug-resistant TB risk factors are likely to differ in low-resource, high HIV prevalence settings due to the increased risk of transmission in congregate settings [17], more rapid progression to active disease following infection [18], and higher mortality from TB/HIV co-infection [4], [19]. Thus, patients are less likely to survive multiple prior courses of TB treatment and fit the classic profile of a “chronic” TB case. Conversely, HIV/AIDS has been associated with TB drug malabsorption [20], which may contribute to higher rates of amplified drug resistance in this setting. To date, there have been no studies of clinical or epidemiologic risk factors for MDR or XDR TB in a high HIV prevalence setting.

We undertook a case-control study in a community with a high prevalence of HIV and drug-resistant tuberculosis in rural South Africa to assess clinical predictors of multi-drug resistant and extensively drug-resistant tuberculosis.

## Methods

### Ethics Statement

The study protocol was approved by the institutional review boards of the Albert Einstein College of Medicine, Yale University, and University of KwaZulu-Natal, and by the KwaZulu-Natal Department of Health. The data used in this study were collected as part of routine medical care in the hospital clinical chart. As these were simply for clinical care, patients were not asked to give informed consent at the time of these clinical encounters. For the purposes of this retrospective study, the requirement for informed consent was waived by the ethics committees listed above, since all data used were previously collected during the course of routine medical care and did not pose any additional risks to the patients.

### Setting

This study was conducted from June 2005 to January 2007 in Tugela Ferry, South Africa, a rural community of approximately 200,000 Zulu people. The case notification rate of tuberculosis in this community is over 1,100 per 100,000 population; MDR and XDR TB incidence were 118 and 72 per 100,000, respectively, in 2007 [4]. Medical services are centered at Church of Scotland Hospital (COSH), a 355-bed government district hospital in Tugela Ferry, where a tuberculosis DOTS program has been in place since 1993. During the time of this study, hospitalized patients resided in congregate 35 to 40-bed wards. HIV prevalence among women seeking antenatal care is estimated at 37%.

During the study period, culture and DST were available for clinicians to order for any tuberculosis suspect, and clinicians were encouraged to obtain these tests for all tuberculosis suspects. This practice differed from other district hospitals in South Africa, and from that recommended by national policy, which recommended restriction of culture and drug-susceptibility testing to TB patients who were failing their current first-line regimen or who were re-treatment cases [21].

Mycobacterial culture was performed at the provincial TB reference laboratory using both liquid (BACTEC mycobacterial growth indicator tube (MGIT)-960 system) and solid media (Middlebrook 7H10), as previously described [7]. Drug susceptibility testing was performed on all positive mycobacterial cultures by the 1% proportional method on Middlebrook 7H10 agar for isoniazid, rifampicin, ethambutol, streptomycin, ciprofloxacin and kanamycin. Pyrazinamide is not included in the standard DST panel, even though it is included in the standard first-line TB treatment regimen.

### Study population

We selected all patients with MDR and XDR TB diagnosed in outpatient clinics or inpatient wards at COSH for whom complete medical records were available for inclusion as cases in the study. A control group of patients with drug-susceptible (DS) TB was selected to achieve a 1∶1∶1 ratio of DS: MDR: XDR TB through review of consecutive patients listed in the TB DOTS office register diagnosed during the same period as cases. In order to fully examine potential risk factors for drug-resistant tuberculosis, patients were not matched by any demographic or clinical variables.

### Data Collection

We collected data from hospital medical records, TB DOTS clinic records, and HIV clinic records. Variables of interest included age, sex, HIV status, CD4 cell counts, antiretroviral therapy (ART) use, extrapulmonary TB, presenting vital signs and laboratory parameters, TB treatment history, and hospitalization at COSH (for any reason) history.

### Statistical Analyses

We compared clinical and demographic variables of patients with DS, MDR and XDR TB by Fisher's exact test for categorical variables and the Student's t-test or Mann-Whitney U test for continuous variables as guided by normality of the data. All three groups were compared against each other (i.e. the MDR TB group and XDR TB group were separately compared against the DS TB group and compared against one another in two-way comparisons).

We calculated odds ratios (OR) and adjusted odds ratios (aOR) using bivariate and multivariable logistic regression to assess independent predictors of MDR TB or XDR TB compared with DS TB controls, as well as XDR TB compared with MDR TB. A cutoff criteria of p-value <0.2 on bivariate analysis was used to select variables for inclusion in the multivariate models comparing MDR TB and XDR TB with DS TB (models 1 and 2 respectively). We performed an Allen-Cady modified, backward selection procedure with gender and age included by default in all models [22]. A cutoff criteria of p-value <0.2 was utilized for termination of variable elimination during backward selection. A third model comparing XDR with MDR TB was constructed using the same variables included in models 1 and 2 for ease of comparison. All tests for significance were two-sided with a p-value <0.05 considered significant. Bivariate and multivariable analyses were performed using Stata (version 10.0).

## Results

### Demographic and Clinical Characteristics

Medical records were reviewed for 116, 123 and 139 patients with DS, MDR and XDR TB, respectively, diagnosed between June 2005 and January 2007. MDR TB patients were resistant to a mean of 2.9 drugs (SD 0.6); XDR TB patients were resistant to a mean of 5.3 drugs (SD 0.8). The most common drug resistance pattern for MDR TB isolates was resistance to isoniazid, rifampicin, and streptomycin (80/123, 65%); the majority of XDR TB isolates were resistant to all six drugs tested: isoniazid, rifampicin, streptomycin, ethambutol, ciprofloxacin and kanamycin (77/139, 55%). There were no differences in the median age of patients in all three groups (Table 1). Female sex was more common among XDR TB patients compared to DS and MDR TB patients (p = 0.03 and p = 0.04, respectively). Approximately one quarter of all patients were diagnosed with extrapulmonary TB in addition to pulmonary TB. The most common form of extrapulmonary TB was pericardial (41% of extrapulmonary cases), followed by lymph node (24%) and pleural (15%).

**Table 1:** Characteristics of patients with drug susceptible, multidrug-resistant and extensively drug-resistant tuberculosis.

	DS	MDR	XDR	MDR vs DS		XDR vs DS	
N	116	123	139	OR	CI	OR	CI
Age, median (IQR)	35 (29–43)	34 (29–43)	34 (29–42)	p = 0.75		p = 1.00	
Female, n (%)	49 (42)	53 (43)	78 (56)	1.04	(0.62–1.73)	1.75	(1.06–2.88)*
Extrapulmonary, n (%)	26 (22)	34 (28)	41 (30)	1.32	(0.73–2.38)	1.45	(0.82–2.56)
Previous TB Treatment							
Past year, n (%)	18 (16)	73 (59)	82 (59)	7.94	(4.28–14.75)*	7.83	(4.27–14.35)*
Ever, n (%)	31 (27)	92 (75)	96 (69)	8.13	(4.56–14.51)*	6.12	(3.54–10.57)*
Previous treatment status known, n (%)	26 (84)	70 (76)	78 (81)				
Previous treatment status: cure, n (%) ϶	19 (73)	17 (24)	15 (19)	0.82	(0.40–1.67)	0.62	(0.30–1.28)
Previous treatment status: default, n (%) ϶	6 (23)	10 (14)	4 (5)	1.62	(0.57–4.62)	0.54	(0.15–1.97)
Previous treatment status: failure, n (%) ϶	1 (4)	43 (61)	59 (76)	61.81	(8.34–458.13)*	84.81	(11.51–624.80)*
Previous Hospitalization							
Past year, n (%)	13 (11)	56 (46)	73 (53)	6.62	(3.36–13.04)*	8.76	(4.50–17.06)*
Ever, n (%)	23 (20)	69 (56)	83 (60)	5.17	(2.90–9.22)*	5.99	(3.39–10.58)*
Hospitalized >14 days, past year, n (%)	4 (3)	32 (26)	43 (31)	9.85	(3.36–28.87)*	12.5	(4.34–36.21)*
Days Hospitalized, past year °							
median (range)	11 (4–36)	17 (6–109)	18 (3–105)	1.12	(1.07–1.17)*	1.14	(1.08–1.19)*
Weight, median (IQR) (kg)	52 (46–57)	48 (45–58)	50 (45–56)	p = 0.32		p = 0.15*	
Hemoglobin, median (IQR) (g/dL)	9.4 (7.7–10.8)	9.8 (7.9–11.5)	9.0 (7.7–10.4)	p = 0.28		p = 0.97	
Albumin, median (IQR) (g/dL)	25.7 (20.8–31.5)	28.0 (7.85–11.45)	21.0 (18.7–25.7)	p = 0.56		p = 0.26	
HIV tested, n (%)	90 (78)	92 (75)	117 (85)				
HIV positive, n (% tested)	78 (87)	85 (92)	115 (98)	1.87	(0.70–4.99)	8.85	(1.93–40.62)*
CD4 Count Available, n (%)†	30 (38)	43 (51)	39 (34)				
Median (IQR)§	110.5 (41–223)	87 (27–222)	60 (26–164)	p = 0.56		p = 0.09*	
<200 cells/mm2	21 (70)	31 (72)	34 (87)	1.11	(0.40–3.09)	2.91	(0.86–9.88)*
ART Before TB Diagnosis, n (%)†	3 (4)	13 (15)	25 (22)	4.51	(1.23–16.50)*	6.94	(2.02–23.90)*
Median duration among treated, days	113	62	120	p = 0.42		p = 0.77	

°Days hospitalized are considered among patients who were hospitalized.

† CD4 cell count available and ART received before TB diagnosis were calculated among the fraction of patients known to have HIV.

§ CD4 cell count was included if it was drawn within 120 days of TB diagnosis.

*Indicates p-value of <0.2 (candidate for multivariable model).

϶Percents calculated from fraction of patients with known retreatment; referent group for ORs was all patients without this retreatment classification (e.g. referent for ‘retreatment without cure’ includes previously untreated patients and those wiith other retreatment class).

The majority of patients with MDR (75%) and XDR TB (69%) had been previously treated for drug-susceptible tuberculosis, whereas only 27% of patients with DS tuberculosis had been treated previously (p<0.0001 and p<0.0001, respectively; Table 1). No patients had undergone prior treatment with second-line drugs. Most previously treated patients in all three groups had been treated within the previous year. Among previously treated patients, most DS TB patients had been cured (73%), while most MDR and XDR TB patients had failed treatment (61% and 76%; p<0.0005 and p<0.0001, respectively).

Patients with MDR or XDR TB were more likely to have been previously hospitalized compared to DS TB controls (p<0.0001 and p<0.0001, respectively; Table 1). Among patients that had been hospitalized in the past year, patients with MDR and XDR TB were hospitalized for more days (median 17 and 18 days, respectively) than those with DS tuberculosis (11 days; p = 0.04 and p = 0.02, respectively).

Patients with XDR TB had a higher prevalence of HIV (98%) than those with MDR TB (92%; p = 0.06) or DS TB (87%; p<0.01). There were no significant differences in median CD4 cell count among the drug resistance groups, though there was a trend towards lower CD4 cell counts among patients with MDR or XDR TB. Among HIV-infected patients, those with MDR and XDR TB were more likely to be receiving antiretroviral therapy at the time of their drug-resistant TB diagnosis (p = 0.02 and p<0.005, respectively).

### Independent predictors of drug resistance

In multivariable analysis comparing MDR or XDR TB cases with DS TB controls, MDR and XDR TB were strongly associated with a history of TB treatment failure (MDR aOR 51.7 [CI 6.6–403.7]; XDR aOR 51.5 [CI 6.4–414.0]) and hospitalization more than 14 days (MDR aOR 3.8 [CI 1.1–13.3], XDR aOR 6.1 [CI 1.8–21.0]; table 2). HIV was an independent risk factor for XDR TB (aOR 8.2, [CI 1.3–52.6]) but not for MDR TB (aOR 1.4 [CI 0.5–4.0]). Antiretroviral therapy and CD4 cell count were not included in the model due to collinearity with HIV.

**Table 2:** Adjusted risk factors for MDR and XDR TB, comparing MDR and XDR to DS TB, then XDR to MDR TB.

			MDR vs DS		XDR vs DS		XDR vs MDR	
			Adjusted OR (95% CI)		Adjusted OR (95% CI)		Adjusted OR (95% CI)	
Hospital admission for >14 days in past year			3.8	(1.1–13.3)	6.1	(1.8–21.0)	1.3	(0.7–2.6)
HIV infected			1.4	(0.5–4.0)	8.2	(1.3–52.6)	5.3	(1.0–27.6)
Previously treated and cured			1.6	(0.7–3.7)	1.1	(0.4–2.6)	0.7	(0.3–1.7)
Previously treated and defaulted			2.5	(0.5–12.3)	1.3	(0.2–8.0)	0.5	(0.1–2.3)
Previously treated and failed treatment			51.7	(6.6–403.7)	51.5	(6.4–414.0)	0.9	(0.4–1.7)

All models are adjusted for Age and Sex (not statistically significant and not shown).

Comparing MDR TB with XDR TB patients in multivariable analysis, the only significant risk factor for XDR TB was HIV infection (aOR 5.3, CI 1.0–27.6).

## Discussion

Recent global data have shown rising rates of drug-resistant TB in sub-Saharan Africa, the region also suffering from the world's highest burden of HIV/AIDS [23]. This is the first study of clinical predictors of MDR and XDR TB in a high HIV prevalence setting and provides important insights into the clinical characteristics of patients with drug-resistant TB. We found that three readily available pieces of clinical data – hospitalization history, TB treatment history and HIV status – were strong independent predictors for MDR or XDR TB. Using these data, clinicians practicing in high HIV prevalence settings may be able to cohort high-risk inpatients to reduce transmission and target drug-susceptibility testing where DST resources are limited. Additionally, our findings support the need for strengthening hospital infection control measures, including reducing the duration of hospitalization in high HIV prevalence settings.

Prior tuberculosis treatment is a well-established risk factor for drug-resistant tuberculosis [15]. Studies from Peru and Russia reported prior treatment among nearly all cases of MDR and XDR TB, with XDR patients having received more courses of therapy than MDR patients [9], [24]. In contrast, nearly 30% of patients in our study had no previous treatment for tuberculosis, and few had received more than one previous treatment regimen. Moreover, there were no patients who had received previous treatment with second-line TB drugs. A recent study of patients being re-treated for TB at Edendale Hospital in KwaZulu-Natal reported an association between treatment failure and any drug resistance [16]; however, only 7% of re-treatment patients in that study had a history of treatment failure. In our study, a history of TB treatment failure was associated with a 50-fold increase in risk of having MDR or XDR TB and was the most common re-treatment status among patients with MDR and XDR TB.

Our study also identified hospitalization as an important risk factor for MDR and XDR tuberculosis, with prolonged hospitalization associated with a 3- to 6-fold increase in risk, respectively. This finding further supports nosocomial transmission as a likely driver of the epidemic in this setting, consistent with previous studies from our site [25], [26]. A recent study suggested that MDR TB patients treated with first line regimens were responsible for the majority of TB transmission on a tuberculosis ward [17]. Delays in the diagnosis of drug resistance and large, congregate TB wards, that are the norm in many high burden settings, remain a perilous combination for transmission of drug-resistant TB.

There has been conflicting evidence about whether HIV is an independent risk factor for primary or acquired drug-resistant tuberculosis [27]–[32]. We found HIV was associated with a markedly greater risk of XDR TB compared with either DS TB or MDR TB. These findings support the notion that HIV-infected patients may be over-represented earlier in epidemics of drug-resistant TB, as immunocompromised individuals are often first to develop clinical disease from recently circulating strains.

Though the numbers were small, an unexpected finding in our study was a significantly higher rate of antiretroviral therapy (ART) use among patients with MDR and XDR TB. This may be explained by the higher rate of previous TB treatment among MDR and XDR TB patients (75% and 69%, respectively) as all TB patients are routinely tested for HIV in Tugela Ferry and initiated on ART within the first few months of treatment. Another possible explanation is that HIV patients receiving ART may have been exposed to other undiagnosed cases of MDR or XDR TB while attending clinic visits. A recent active TB case-finding study at our site identified high rates of undiagnosed MDR and XDR TB among patients attending the HIV clinic (NS Shah, unpublished data). Further molecular epidemiology studies are needed to better define transmission patterns in hospital, clinic, and community settings in Tugela Ferry. Nonetheless, improved efforts to actively screen for TB and strengthen infection control in HIV care and treatment facilities are needed to avoid undermining gains achieved by ART roll-out programs [33], [34].

Few studies have compared patients with MDR and XDR TB to determine whether they differ in clinical or demographic characteristics. A report from Estonia, a country with low HIV prevalence, found that they shared common predictor variables in comparison to drug-susceptible TB, though direct comparisons were not made [35]. In the high HIV prevalence setting in the current study, there were few relevant clinical differences between patients upon diagnosis, suggesting that control efforts – such as improving TB treatment adherence and infection control measures – would be effective against both the MDR and XDR TB epidemics.

These data must be interpreted in the context of the study design and setting. First, this was a hospital-based study and may reflect severity of disease and risk factors for TB patients diagnosed at a hospital or at hospital-based clinics. Second, the prevalence of MDR and XDR TB is higher in Tugela Ferry than that reported from other districts in South Africa. The contribution of various risk factors – such as hospitalization – is likely closely related to the prevalence of drug resistance in the hospital and community. However, the more widespread use of culture and DST in Tugela Ferry provides a more representative sample of the true MDR and XDR TB epidemic. As other high HIV prevalence communities undertake similarly representative surveys, as recently performed in Khayelitsha, South Africa, high drug-resistant TB prevalence rates, similar to ours, may be uncovered [36].

Third, because DST results from the first TB episode were not available on most re-treatment patients in our study, it is not possible to know whether previous TB treatment was a risk factor due to acquisition of drug-resistance or exogenous infection (or re-infection) with drug-resistant strains. However, our prior studies have found exogenous re-infection with drug-resistant strains to be an important mechanism in this setting [25]. Moreover, because no patients had received second-line TB drugs, XDR TB among patients who were previously treated clearly represents primary transmission of drug resistance.

Lastly, we did not evaluate certain demographic and clinical variables identified in other studies as risk factors for MDR or XDR TB – such as homelessness, alcohol use, and imprisonment [35], [37], [38] – due to the lack of rigorous documentation of these data in medical records. While imprisonment and homeless rates are low in this community, alcohol use may be an important factor.

Although there has been renewed enthusiasm for the development and roll-out of low-cost, rapid drug-susceptibility tests for TB, the majority of patients in resource-limited settings do not have access to these new tests or even conventional drug-susceptibility testing, even in settings such as South Africa with a relatively advanced laboratory infrastructure [39]. Delays in diagnosis lead to clinical deterioration of patients and ongoing drug-resistant TB transmission in the community or hospital; in a high HIV prevalence setting, this can have disastrous consequences. In the absence of universal culture and drug-susceptibility testing for all TB patients, our study provides an evidence base for better identifying high-risk patients for targeted drug-susceptibility testing, isolation, and empiric use of second-line TB drugs while awaiting results.

## References

[1] Seung KJ, Omatayo DB, Keshavjee S, Furin JJ, Farmer PE (2009). Early outcomes of MDR-TB treatment in a high HIV-prevalence setting in Southern Africa.. PLoS ONE.

[2] Calver AD, Falmer AA, Murray M, Strauss OJ, Streicher EM (2010). Emergence of increased resistance and extensively drug-resistant tuberculosis despite treatment adherence, South Africa.. Emerging Infect Dis.

[3] Hassim S, Shaw PA, Sangweni P, Malan L, Ntshani E (2010). Detection of a substantial rate of multidrug-resistant tuberculosis in an HIV-infected population in South Africa by active monitoring of sputum samples.. Clin Infect Dis.

[4] Gandhi NR, Shah NS, Andrews JR, Vella V, Moll AP (2010). HIV coinfection in multidrug- and extensively drug-resistant tuberculosis results in high early mortality.. Am J Respir Crit Care Med.

[5] Dheda K, Shean K, Zumla A, Badri M, Streicher EM (2010). Early treatment outcomes and HIV status of patients with extensively drug-resistant tuberculosis in South Africa: a retrospective cohort study.. Lancet.

[6] World Health Organization (2009). Global Tuberculosis Control: A short update to the 2009 report..

[7] Gandhi NR, Moll A, Sturm AW, Pawinski R, Govender T (2006). Extensively drug-resistant tuberculosis as a cause of death in patients co-infected with tuberculosis and HIV in a rural area of South Africa.. Lancet.

[8] Basu S, Friedland GH, Medlock J, Andrews JR, Shah NS (2009). Averting epidemics of extensively drug-resistant tuberculosis.. Proc Natl Acad Sci USA.

[9] Mitnick CD, Shin SS, Seung KJ, Rich ML, Atwood SS (2008). Comprehensive treatment of extensively drug-resistant tuberculosis.. N Engl J Med.

[10] Mitnick C, Bayona J, Palacios E, Shin S, Furin J (2003). Community-based therapy for multidrug-resistant tuberculosis in Lima, Peru.. N Engl J Med.

[11] Solari L, Acuna-Villaorduna C, Soto A, Agapito J, Perez F (2008). A clinical prediction rule for pulmonary tuberculosis in emergency departments.. Int J Tuberc Lung Dis.

[12] Novelli EM, Hittner JB, Davenport GC, Ouma C, Were T (2010). Clinical predictors of severe malarial anaemia in a holoendemic Plasmodium falciparum transmission area.. Br J Haematol.

[13] Costello C, Nelson KE, Jamieson DJ, Spacek L, Sennun S (2005). Predictors of low CD4 count in resource-limited settings: based on an antiretroviral-naive heterosexual thai population.. J Acquir Immune Defic Syndr.

[14] Mwaniki MK, Nokes DJ, Ignas J, Munywoki P, Ngama M (2009). Emergency triage assessment for hypoxaemia in neonates and young children in a Kenyan hospital: an observational study.. Bull World Health Organ.

[15] Vashakidze L, Salakaia A, Shubladze N, Cynamon M, Barbakadze K (2009). Prevalence and risk factors for drug resistance among hospitalized tuberculosis patients in Georgia.. Int J Tuberc Lung Dis.

[16] Schreiber Y, Herrera A, Wilson D, Wallengren K, Draper R (2009). Tuberculosis retreatment category predicts resistance in hospitalized retreatment patients in a high HIV prevalence area.. Int J Tuberc Lung Dis.

[17] Escombe AR, Moore DAJ, Gilman RH, Pan W, Navincopa M (2008). The infectiousness of tuberculosis patients coinfected with HIV.. PLoS Med.

[18] Selwyn PA, Hartel D, Lewis VA, Schoenbaum EE, Vermund SH (1989). A prospective study of the risk of tuberculosis among intravenous drug users with human immunodeficiency virus infection.. N Engl J Med.

[19] Brust JCM, Gandhi NR, Carrara H, Osburn G, Padayatchi N (2010). High treatment failure and default rates for patients with multidrug-resistant tuberculosis in KwaZulu-Natal, South Africa, 2000-2003.. Int J Tuberc Lung Dis.

[20] Patel KB, Belmonte R, Crowe HM (1995). Drug malabsorption and resistant tuberculosis in HIV-infected patients.. N Engl J Med.

[21] South Africa Department of Health (2004). The South African National Tuberculosis Control Programme Practical Guidelines.

[22] Vittinghoff E, Glidden D, Shiboski S, McCulloch C (2004). Regression Methods in Biostatistics..

[23] World Health Organization. (2010). Multidrug and Extensively Drug-Resistant TB (M/XDR-TB): 2010 Global Report on Surveillance and Response..

[24] Keshavjee S, Gelmanova I, Farmer P, Mishustin S, Strelis A (2008). Treatment of extensively drug-resistant tuberculosis in Tomsk, Russia: a retrospective cohort study.. Lancet.

[25] Andrews JR, Gandhi NR, Moodley P, Shah NS, Bohlken L (2008). Exogenous reinfection as a cause of multidrug-resistant and extensively drug-resistant tuberculosis in rural South Africa.. J Infect Dis.

[26] Basu S, Andrews JR, Poolman EM, Gandhi NR, Shah NS (2007). Prevention of nosocomial transmission of extensively drug-resistant tuberculosis in rural South African district hospitals: an epidemiological modelling study.. Lancet.

[27] Churchyard GJ, Corbett EL, Kleinschmidt I, Mulder D, De Cock KM (2000). Drug-resistant tuberculosis in South African gold miners: incidence and associated factors.. Int J Tuberc Lung Dis.

[28] Punnotok J, Shaffer N, Naiwatanakul T, Pumprueg U, Subhannachart P (2000). Human immunodeficiency virus-related tuberculosis and primary drug resistance in Bangkok, Thailand.. Int J Tuberc Lung Dis.

[29] Campos PE, Suarez PG, Sanchez J, Zavala D, Arevalo J (2003). Multidrug-resistant Mycobacterium tuberculosis in HIV-infected persons, Peru.. Emerging Infect. Dis.

[30] Gordin FM, Nelson ET, Matts JP, Cohn DL, Ernst J (1996). The impact of human immunodeficiency virus infection on drug-resistant tuberculosis.. Am J Respir Crit Care Med.

[31] Espinal MA, Laserson K, Camacho M, Fusheng Z, Kim SJ (2001). Determinants of drug-resistant tuberculosis: analysis of 11 countries.. Int J Tuberc Lung Dis.

[32] Suchindran S, Brouwer ES, Van Rie A (2009). Is HIV infection a risk factor for multi-drug resistant tuberculosis? A systematic review.. PLoS ONE.

[33] Andrews JR, Shah NS, Gandhi N, Moll T, Friedland G (2007). Multidrug-resistant and extensively drug-resistant tuberculosis: implications for the HIV epidemic and antiretroviral therapy rollout in South Africa.. J Infect Dis.

[34] Shenoi SV, Escombe AR, Friedland G (2010). Transmission of drug-susceptible and drug-resistant tuberculosis and the critical importance of airborne infection control in the era of HIV infection and highly active antiretroviral therapy rollouts.. Clin Infect Dis.

[35] Kliiman K, Altraja A (2009). Predictors of extensively drug-resistant pulmonary tuberculosis.. Ann Intern Med.

[36] Cox HS, McDermid C, Azevedo V, Muller O, Coetzee D (2010). Epidemic levels of drug resistant tuberculosis (MDR and XDR-TB) in a high HIV prevalence setting in Khayelitsha, South Africa.. PLoS ONE.

[37] Casal M, Vaquero M, Rinder H, Tortoli E, Grosset J (2005). A case-control study for multidrug-resistant tuberculosis: risk factors in four European countries.. Microb Drug Resist.

[38] Drobniewski F, Balabanova Y, Nikolayevsky V, Ruddy M, Kuznetzov S (2005). Drug-resistant tuberculosis, clinical virulence, and the dominance of the Beijing strain family in Russia.. JAMA.

[39] Dowdy DW, Chaisson RE, Maartens G, Corbett EL, Dorman SE (2008). Impact of enhanced tuberculosis diagnosis in South Africa: a mathematical model of expanded culture and drug susceptibility testing.. Proc Natl Acad Sci USA.

